# Angiotensin II stimulates sympathetic neurotransmission to adipose tissue

**DOI:** 10.1002/phy2.14

**Published:** 2013-06-24

**Authors:** Victoria L King, Victoria L English, Kalyani Bharadwaj, Lisa A Cassis

**Affiliations:** 1 Division of Cardiology, University of KentuckyLexington, Kentucky, 40536; 2 Department of Molecular and Biomedical Pharmacology, University of KentuckyLexington, Kentucky, 40536; 3 Piramal Life Sciences LimitedMumbai, India

**Keywords:** Body weight, catecholamine turnover, norepinephrine

## Abstract

Angiotensin II (AngII) facilitates sympathetic neurotransmission by regulating norepinephrine (NE) synthesis, release, and uptake. These effects of AngII contribute to cardiovascular control. Previous studies in our laboratory demonstrated that chronic AngII infusion decreased body weight of rats. We hypothesized that AngII facilitates sympathetic neurotransmission to adipose tissue and may thereby decrease body weight. The effect of chronic AngII infusion on the NE uptake transporter and NE turnover was examined in metabolic (interscapular brown adipose tissue, ISBAT; epididymal fat, EF) and cardiovascular tissues (left ventricle, LV; kidney) of rats. To examine the uptake transporter saturation isotherms were performed using [^3^H]nisoxetine (NIS). At doses that lowered body weight, AngII significantly increased ISBAT [^3^H]NIS binding density. To quantify NE turnover, alpha-methyl-para-tyrosine (AMPT) was injected in saline-infused, AngII-infused, or saline-infused rats that were pair-fed to food intake of AngII-infused rats. AngII significantly increased the rate of NE decline in all tissues compared to saline. The rate of NE decline in EF was increased to a similar extent by AngII and by pair feeding. In rats administered AngII and propranolol, reductions in food and water intake and body weight were eliminated. These data support the hypothesis that AngII facilitates sympathetic neurotransmission to adipose tissue. Increased sympathetic neurotransmission to adipose tissue following AngII exposure is suggested to contribute to reductions in body weight.

## Introduction

Angiotensin II (AngII), the primary peptide of the renin–angiotensin system (RAS) with biologic activity, is important in the regulation of arterial pressure and fluid and electrolyte balance (Peach 1981). On the basis of a variety of evidence, including demonstration of components necessary for AngII synthesis, AngII receptor localization, detectable quantities of immunoreactive AngII, and functional responsiveness to AngII, local tissue RAS have been proposed (Phillips et al. 1993). Previous studies in our laboratory demonstrated a high level of angiotensinogen mRNA expression (Cassis et al. 1988a), renin-like activity (Shenoy and Cassis 1997), AngII receptor localization (Cassis et al. 1998a), immunoreactive AngII (Dwoskin et al. 1992; Shenoy and Cassis 1997), and AngII regulation of sympathetic neurotransmission (Dwoskin et al. 1992; English and Cassis 1999) in rat adipose tissue. Moreover, a variety of evidence supports the existence of a local RAS in rodent and human adipocytes (Cassis et al. 2008; Thatcher et al. 2009).

The ability of adipocytes to synthesize components of the RAS raises the question of whether AngII acts locally to influence adipose tissue function. Recent studies from our laboratory demonstrated that deficiency of angiotensin type 1a receptors (AT1aR) in adipocytes of mice resulted in adipocyte hypertrophy in lean, but not in obese mice (Putnam et al. 2012). These effects were linked to reductions in differentiation of adipocytes (Putnam et al. 2012), suggesting that AngII influences fat mass. Indeed, infusion of AngII to rats (Brink et al. 1996; Cassis et al. 1998b; English and Cassis 1999) and mice (Brink et al. 2001; Rezk et al. 2012) decreases body weight, food intake, and adipose tissue mass. These effects of AngII appear to be independent of elevations in blood pressure (Brink et al. 1996; Cassis et al. 1998a; English and Cassis 1999).

A well known effect of AngII actions at AT1 receptors is facilitation of the sympathetic nervous system by increasing the synthesis (Yang and Raizada 1998), release (Kawasaki et al. 1982; Meldrum et al. 1984; Kurz et al. 1997; Storgaard and Nedergaard 1997; English and Cassis 1999), and uptake of norepinephrine (NE) (Yu et al. 1996; Yang and Raizada 1998). The ability of AngII to facilitate sympathetic nervous system has been demonstrated in the brain (Meldrum et al. 1984), resistance vessels (Kawasaki et al. 1982), heart (Ramchandra et al. 2012), and the kidney (Burke et al. 2007), important sites in cardiovascular control. Previous studies in our laboratory demonstrated that exogenous and endogenous AngII facilitates the evoked in vitro release of NE from slices of interscapular brown adipose tissue (ISBAT) (Cassis 1993, 1994), a site demonstrated to express components of the RAS (Cassis et al. 1988a,b; Cassis 1994). Additional studies demonstrated that at infusion doses of AngII capable of decreasing body weight in rats, the in vitro effect of AngII to enhance sympathetic neurotransmission to ISBAT was increased (English and Cassis 1999) and rats exhibited increased oxygen consumption (Cassis et al. 2002). Collectively, these results suggest that a portion of AngII-mediated reductions in body weight arise from increased sympathetic activity for the control of lipid metabolism and energy expenditure.

In this study, we tested the hypothesis that AngII facilitates sympathetic neurotransmission to adipose tissue and may thereby decrease body weight. To test this hypothesis, we infused rats chronically with AngII and examined integral components of the sympathetic nervous system including the NE neuronal uptake transporter and the turnover of catecholamines in tissues with metabolic (ISBAT; epididymal fat, EF) and cardiovascular (left ventricle, LV; kidney) relevance. Moreover, we determined the role of metabolic status in the effect of AngII by examining NE turnover in rats pair-fed to food intake levels of AngII-infused rats. Finally, we determined the effect of sympathetic blockade with the nonselective β-receptor antagonist, propranolol, on AngII-mediated reductions in body weight.

## Materials and Methods

### AngII infusion model

Male, Sprague-Dawley rats (350–400 g; Harlan Laboratories, IN) were used in all studies. All rats were housed 2/cage in an approved animal facility for 1 week before use under a 12 h light/dark cycle and were given free access to standard rat laboratory diet and water. During each experimental protocol the rats were housed individually in cages for the daily (10:00 am) measurement of body weight, food, and water intake. Baseline measurements of food intake, water intake, and body weight were taken for 3 days preceding and throughout each experimental protocol. Food intake was quantified by weighing food provided to the cage and that remaining 24 h later, with the difference daily food intake. Water intake was quantified by weighing water bottles on a daily basis. All studies were reviewed and approved by the Institutional Animal Care and Use Committee.

For AngII infusion, rats were shaved in the interscapular region, and osmotic minipumps (Alzet Model 2001 for 7 day infusion; Model 2002 for 14 day infusion, Durect, CA) were implanted subcutaneously. Minipumps contained either AngII (200–600 ng/kg per minute; Sigma, St. Louis, MO) or sterile saline (sham-surgery) and were primed according to the manufacturer's instructions preceding implantation to assure immediate subcutaneous delivery of AngII. The skin overlaying the minipump was closed with surgical staples, and the rats were allowed to recover on warmed heating pads.

In pair-feeding studies, three groups of rats were examined for 14 days: saline-infused, AngII-infused (400 ng/kg per minute), and saline-infused rats receiving the food intake level of rats in the AngII-infusion group. For these studies, the average daily food intake of AngII-infused rats over a 24 h period was given to pair-fed rats on the subsequent day.

In studies with propranolol, four groups of rats were examined for 14 days: saline-infused, saline + propranolol (9.9 mg/kg per day), AngII-infused (400 ng/kg per min), AngII + propranolol. For 3 days prior to osmotic minipump implantation, rats designated to the propranolol groups were pretreated with propranolol in the drinking water at a dose of 9.9 mg/kg per day. Thereafter, propranolol (9.9 mg/kg per day) was infused via the osmotic minipump.

At study endpoint, rats were anesthetized (ketamine/xylazine, 40 and 3 mg/kg, respectively, i.p.) for exsanguination and tissue harvest. Blood was collected into prechilled tubes containing EDTA (0.2 mol/L) to obtain plasma after centrifugation at 4°C. EF, ISBAT, LV, or kidney were removed and either snap frozen for quantification of catecholamines, or processed as described below for quantification of NE uptake transporter binding kinetics.

### Systolic blood pressure

Systolic pressure was measured by use of a Narco system including an inflatable tail cuff, a pressure and pulse transducer, and a recording polygraph. The systolic pressure from three separate measurements was averaged from each rat. Systolic pressure was recorded on the day of implantation of osmotic minipumps (baseline) and on the final day of the study.

### [^3^H] Nisoxetine binding in ISBAT, LV

[^3^H] Nisoxetine (NIS) binding was measured in membranes prepared from saline- and AngII (200–600 ng/kg per minute for 14 days)-infused rats according to previously published methods (King et al. 1999). The uptake inhibitor, NIS was used as a selective ligand for the NE uptake transporter (Tejani-Butt 1992). Tissues were removed, placed in ice-cold buffer (5 mL; 50 mmol/L Tris, 120 mmol/L NaCl, 5 mmol/L KCl, pH 7.4) and homogenized three times for 10 sec with a polytron (Kinematica GmbH, Kinematica AG, Luzerne, Switzerland). Homogenates were diluted to a total volume of 30 mL with ice-cold membrane buffer and centrifuged at 1100*g* for 15 min at 4°C to remove tissue debris. The supernatant from this centrifugation was diluted to 30 mL using fresh buffer and centrifuged at 40,000*g* for 10 min at 4°C to obtain the membrane pellet. The centrifugation procedure was repeated and the final membrane pellet (1–2.5 mg protein/mL buffer) was resuspended in binding buffer (50 mmol/L Tris, 300 mmol/L NaCl, 5 mmol/L KCl, pH 7.4). Protein concentration was determined spectrophotometrically using coomassie blue dye with bovine serum albumin as the standard (Bradford 1976).

Saturation binding isotherms were performed by incubating duplicate aliquots of membrane (70 μg) with an increasing concentration of [*n*-methyl-^3^H]NIS (70 Ci/mmol specific activity; New England Nuclear, Boston, MA) (0.1–20 nmol/L, 8 points, 50 μL) and binding buffer (250 μL) for 30 min at 22°C. Nonspecific binding was determined at each concentration of [^3^H]NIS by the addition of mazindol (2 μmol/L; RBI Natick, MA). Binding was terminated by filtration over presoaked (0.3% polyethyleneimine) glass microfiber filters (#32; Schleicher & Schuell Keene, NH) using a Brandel cell harvester. Radioactivity retained on the filters was measured by liquid scintillation spectrometry. Saturation isotherms for specific [^3^H]NIS binding were constructed using GraphPad Prism. For the determination of *K*d and *B*max, data were analyzed by nonlinear regression analysis using LIGAND (McPherson 1983).

### Measurement of NE turnover

Measurement of NE turnover in tissues was according to previously published methods (Brodie et al. 1966; Cassis et al. 1988c; Brito et al. 2008). Rats designated to control (saline-infused), AngII (400 ng/kg per minute), or pair-fed groups were injected (i.p.) with either vehicle or the tyrosine hydroxylase inhibitor, α-methyl-*p*-tyrosine methyl ester (AMPT, 300 mg/kg; Sigma, St. Louis, MO) either once or twice (second injection at 6 h in rats designated to the 8 h time point) on the final day of the study. At designated times (0, 4, or 8 h) after AMPT injection, rats were anesthetized with ketamine/xylazine (40 and 3 mg/kg, respectively, i.p.), blood was obtained by aortic puncture and tissues (ISBAT, LV, EF, kidney) were removed and frozen. The NE concentration in plasma and tissues was determined using high-performance liquid chromatography (HPLC) with electrochemical detection by previously published methods (King et al. 1999). For tissue NE concentration, the entire organ was removed and processed through alumina extraction with an internal standard (dihydroxybenzylamine) to correct for recovery (>90%). An aliquot of the alumina tissue extract was injected onto the HPLC using electrochemical detection to quantify catecholamines (NE concentration normalized to the tissue wet weight). Linear regression of the log[NE] versus time was performed using individual data points obtained at 0, 4, and 8 h after tyrosine hydroxylase inhibition. The slope (m) and standard error of the regression coefficient were computed by covariance matrix analysis. Between group comparison of slopes was performed using covariance matrix analysis. The rate constant for NE disappearance (k[hr^-1^]; defined as m/0.434) and the NE turnover time (1/k_-1_) were calculated as described by Brodie et al. (1966).

### Measurement of plasma free fatty acid concentrations

Plasma free fatty acid concentrations were quantified using the Wako NEFA C enzymatic kit (Wako Diagnostics, Richmond, VA) in rats infused with different infusion doses of saline or AngII or in rats infused with AngII in the absence or presence of propranolol.

### Statistical analysis

For dose–response data (body weight, food, and water intake) for AngII infusion, a two-way analysis of variance (ANOVA) with time as a repeated measure was performed followed by Tukey–Kramer multiple comparisons test for post hoc analysis. For [^3^H]NIS binding parameters (*B*max, *K*d), a one-way ANOVA was performed. For NE turnover studies, NE tissue concentration was converted to log, plotted against time, and linear regression performed. The slope of the line was used to calculate the rate constant for NE decline. Regression lines were analyzed by covariance matrix analysis for significant difference between groups.

## Results

### Chronic AngII infusion decreases body weight, elevates systolic blood pressure, and increases plasma NE concentrations

**Figure 1:**
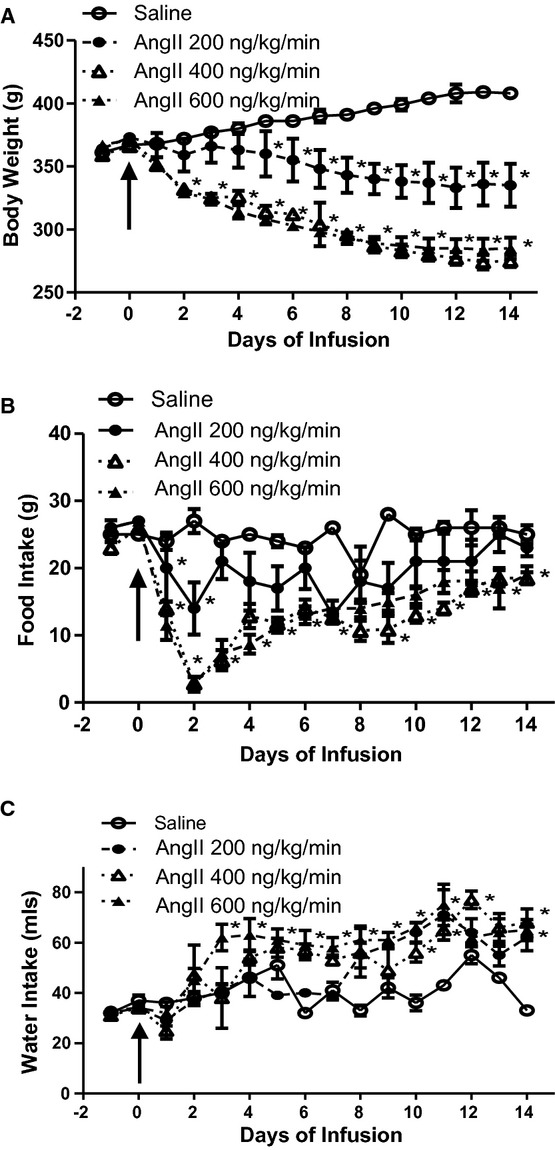
Chronic angiotensin II (AngII) infusion dose-dependently regulates body weight (A), food intake (B), and water intake (C). Baseline measurements of body weight, food and water intake were taken for 3 days prior to osmotic minipump implantation (arrow). Rats were administered either saline or AngII (200–600 ng/kg per minute) for 14 days by osmotic minipump. Measurements were taken daily at 10:00 am. Data are mean ± SEM from *n* = 8 rats/group; “*” denotes significantly different from saline.

We performed dose–response studies infusing AngII (200–600 ng/kg per minute) or saline to rats for 14 days. At 200 ng/kg per minute of AngII, body weight was significantly decreased compared to saline on days 5–14 (Fig. 1A). At higher doses (400, 600 ng/kg per minute) of AngII, body weight was significantly decreased compared to saline on days 2–14 (Fig. 1A). Food intake was significantly decreased compared to saline at all doses of AngII on days 1–3 (Fig. 1B). In all rats infused with AngII, food intake gradually returned toward control in a dose- and time-dependent manner. However, body weight continued to decline. Reductions in food intake and body weight in response to 400 and 600 ng/kg per minute of AngII were significantly greater than those observed at 200 ng/kg per minute. The magnitude of the reduction in food intake and body weight was similar at doses of 400 and 600 ng/kg per minute of AngII, demonstrating that maximal responses had occurred. The weight of EF, but not LV was significantly reduced by infusion of AngII (Table 1; *P* < 0.05). Water intake was significantly increased by all doses of AngII (Fig. 1C; *P* < 0.05).

**Table 1:** Characteristics of rats infused with different doses of AngII

	Saline	AngII (200 ng/kg per minute)	AngII (400 ng/kg per minute)	AngII (600 ng/kg per minute)
Systolic blood pressure (mmHg)	88 ± 7	134 ± 6*	98 ± 25	120 ± 7*
Left ventricle (g)	0.93 ± 0.07	1.17 ± 0.09	1.17 ± 0.09	0.97 ± 0.07
Epididymal adipose (g)	3.73 ± 0.33	4.00 ± 0.17	2.70 ± 0.26*	1.80 ± 0.26*

Data are mean ± SEM from 3 to 10 rats/group.

* *P* < 0.05 compared to saline.

**Figure 2:**
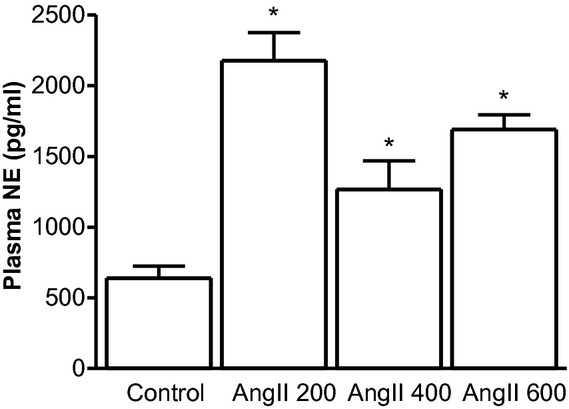
Chronic angiotensin II (AngII) infusion increases plasma norepinephrine (NE concentration. AngII (200–600 ng/kg per minute) or saline were infused for 14 days by osmotic minipump. NE in plasma was extracted over alumina, followed by high-performance liquid chromatography (HPLC) with electrochemical detection for separation and quantification. Data are mean ± SEM from *n* = 8 rats/group; “*” denotes significantly different from control.

Systolic blood pressure was significantly increased by AngII infusion (Table 1). Plasma NE concentration was significantly increased in rats infused with all doses of AngII compared to control (Fig. 2). However, the magnitude of the increase in plasma NE concentration was greatest at the lowest dose of AngII (200 ng/kg per minute).

### Chronic AngII infusion increases NE transporter density in brown adipose tissue

**Figure 3:**
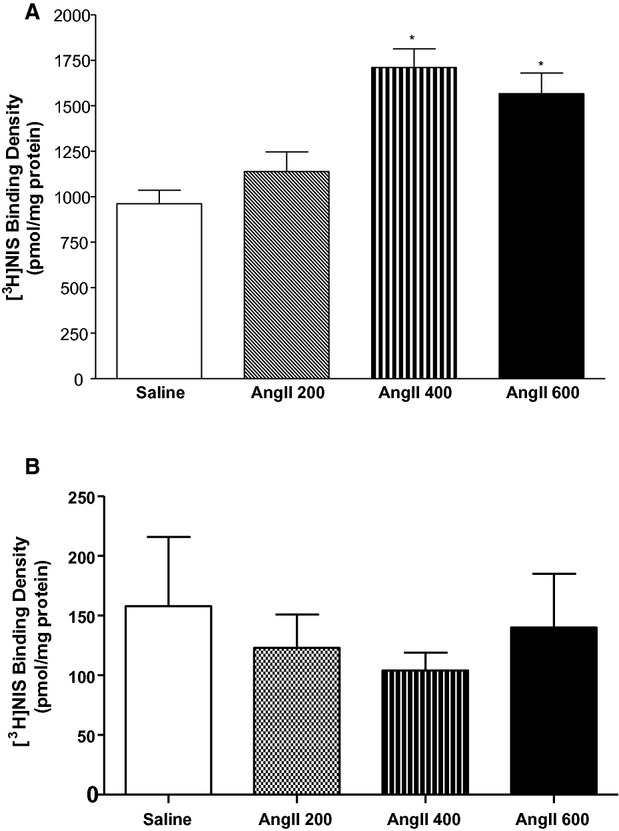
[^3^H]Nisoxetine (NIS) binding density is increased in brown adipose tissue, but not in left ventricle from angiotensin II (AngII)-infused rats. Rats were administered saline- or AngII (200–600 ng/kg per minute) for 14 days. ISBAT (A) and LV (B) were removed and membranes prepared for saturation isotherm binding using [^3^H] NIS as a ligand for the norepinephrine (NE) uptake transporter as described in methods. Data are mean ± SEM from *n* = 8 rats/dose of AngII; “*” denotes significantly different from saline-infused rats (*P* < 0.05).

Saturation isotherms for [^3^H]NIS binding were performed in ISBAT and LV from saline or AngII-infused rats. In ISBAT membranes from all rats, [^3^H]NIS binding was saturable, of high affinity (nmol/L) and to a single class of sites. There was no effect of AngII infusion on the affinity of [^3^H]NIS binding (data not shown). Infusion of AngII resulted in a dose-dependent significant increase in the density of [^3^H]NIS binding sites in ISBAT membranes (Fig. 3A; *P* < 0.05). At 400 ng/kg per minute of AngII, [^3^H]NIS binding density in ISBAT was increased by 77%. At 600 ng/kg per minute of AngII infusion, [^3^H]NIS binding density in ISBAT was significantly increased (by 63%) compared to control, but was not increased compared to 400 ng/kg per minute. In LV, [^3^H]NIS bound to a single class of sites (data not shown). Moreover, in tissues from saline rats, the density of [^3^H]NIS binding sites was 9.5-fold lower in LV than ISBAT (Fig. 3A and B). Chronic infusion of AngII at any dose did not significantly alter [^3^H]NIS binding density (Fig. 3B) or affinity (data not shown) in LV compared to saline.

### Chronic AngII infusion increases NE turnover in brown and white adipose tissue

The purpose of this study was to determine the effect of chronic AngII infusion on the turnover of NE in tissues with metabolic and cardiovascular relevance to the effects of AngII. AngII (400 ng/kg per minute) or saline (control) were infused for either 7 (*n* = 5 rats/group/time point after AMPT injection) or 14 days (*n* = 5 rats/group/time point after AMPT). For studies at 14 days, a third group of rats (*n* = 5 rats/time point after AMPT) that were pair-fed to the food intake of AngII-infused rats were included. On the final day, rats from each group were injected with AMPT and killed at different time points for measurement of tissue NE decline.

Following 7 days of AngII infusion, blood pressure was significantly increased (saline: 105 ± 8; AngII, 137 ± 13 mmHg, *P* < 0.05) and body weight was significantly decreased (saline: 367 ± 6; AngII: 302 ± 9 g, *P* < 0.05) compared to saline and compared to starting body weight (data not shown). Plasma NE concentrations were significantly increased in AngII-infused rats compared to saline (saline: 453 ± 75; AngII: 1804 ± 445 pg/mL, *P* < 0.05). The rate of NE decline (*k*) was not significantly influenced in any organ following 7 days of AngII infusion (data not shown); however, NE turnover rate (*k* × NE_0_) was significantly decreased in LV from AngII-infused rats compared to saline (saline: 64 ± 3; AngII: 23 ± 3, *P* < 0.05).

**Figure 4:**
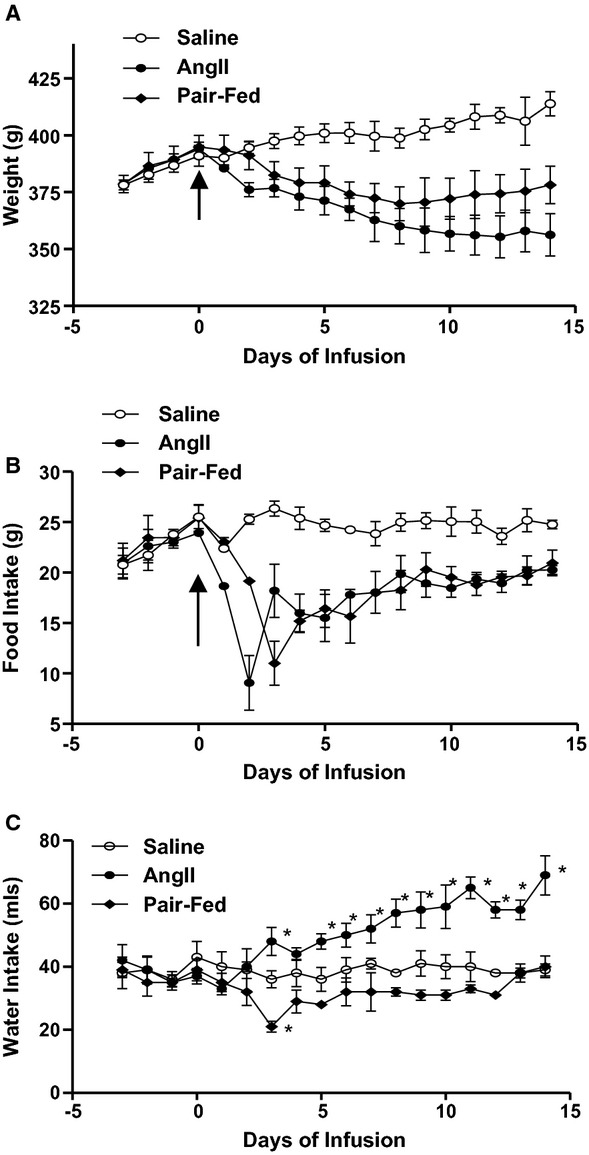
Pair feeding demonstrates that reductions in food intake partially mediate body weight reductions of angiotensin II (AngII)-infused rats. Rats were administered saline, AngII (400 ng/kg per minute) or were pair-fed to food intake of AngII-infused rats for 14 days. Body weight (A), food intake (B), and water intake (C) of rats in each group. Data are mean ± SEM from *n* = 15 rats/group. *Denotes significantly different from saline-infused rats (*P* < 0.05).

**Figure 5:**
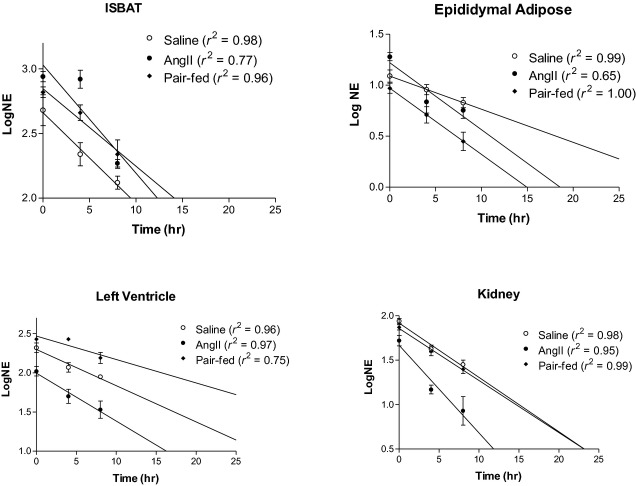
Decline of norepinephrine (NE) in tissues after synthesis inhibition. Rats were administered saline, angiotensin II (AngII; 400 ng/kg per minute), or were pair-fed to the food intake of AngII-infused rats for 14 days. On the final day, rats were injected with alpha-methyl-para-tyrosine (AMPT) and tissues removed for determination of NE concentration by high-performance liquid chromatography (HPLC). The logNE concentration in tissues from rats in each group was plotted against the time after AMPT administration. Linear regression was used to determine the slope of the line for the calculation of the rate of NE decline (*k* = slope/0.434). The correlation coefficient (*r*^2^) from the regression analysis of each line is provided with the symbol for the respective groups.

**Figure 6:**
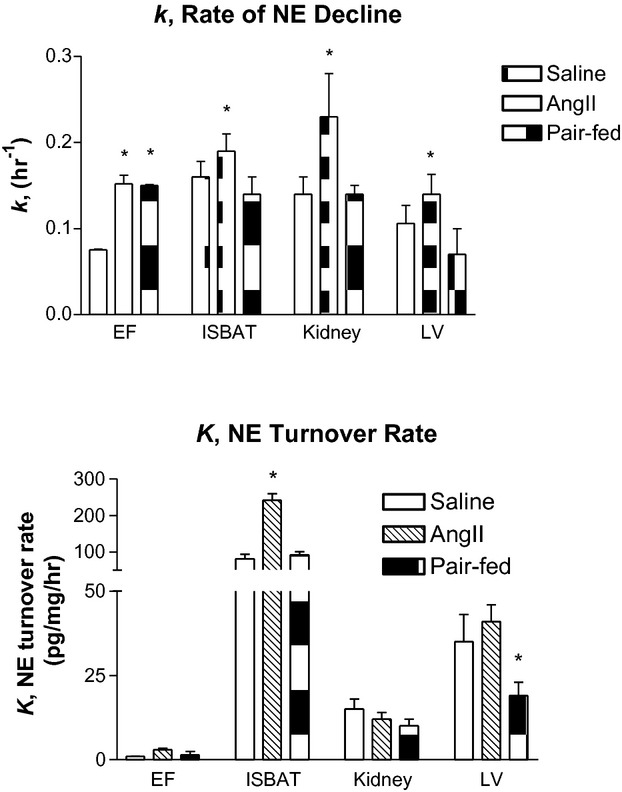
Angiotensin II (AngII) infusion increases norepinephrine (NE) turnover in brown adipose tissue. Top: *k*, the rate of NE decline. Linear regression analysis was performed on data illustrated in Figure 5 to calculate the rate of NE decline (*k* = slope/0.434). Bottom: *K*, NE turnover rate (endogenous NE concentration × *k*) was increased in ISBAT from AngII-infused rats, and decreased in LV from pair-fed rats compared to saline controls. Data are mean ± SEM from *n* = 5 rats/group/time point; “*” denotes significantly different from saline, *P* < 0.05.

Following 14 days of AngII infusion, blood pressure was significantly increased compared to saline and pair-fed rats (saline: 108 ± 9; pair-fed: 105 ± 4; AngII: 132 ± 5 mmHg; *P* < 0.05). Body weight was significantly decreased in AngII-infused and pair-fed rats compared to saline (Fig. 4A), with reductions in food intake (Fig. 4B) accounting for approximately 64% of the effect of AngII on body weight at 14 days. Water intake was significantly increased in AngII-infused rats compared to saline and pair-fed rats (Fig. 4C). The endogenous NE concentration in LV and kidney from AngII-infused rats was significantly decreased compared to saline and pair-fed rats (Table 2). In contrast, AngII infusion resulted in a significant increase in NE concentration in ISBAT and EF compared to saline and pair-fed rats. In tissues from rats in each group the log NE concentration was regressed against time after AMPT administration and covariance matrix analysis of regression curves was performed to determine significant differences between groups (Fig. 5). The rate of NE decline (*k*) was determined from the slope of regression lines (Fig. 5) and was significantly greater in LV, kidney, and ISBAT from AngII-infused rats compared to saline and pair-fed rats (Figs. 5 and 6A). In EF, the rate of NE decline was increased to a similar extent in AngII-infused and pair-fed rats. The NE turnover rate (*K*) was significantly increased in ISBAT from AngII-infused rats compared to saline and pair-fed rats; in contrast, in LV the NE turnover rate was decreased in pair-fed rats compared to saline and AngII-infused rats (Fig. 6B).

**Table 2:** Endogenous tissue NE concentration

	Saline	AngII	Pair-fed
EF	12 ± 1.9	19 ± 1.5^1^	9 ± 1.2^2^
ISBAT	562 ± 79	885 ± 76^1^	668 ± 61
LV	210 ± 27	104 ± 12^1^	269 ± 14^2^
Kidney	92 ± 6	53 ± 5^1^	74 ± 5^2^

Values are pg/mg wet weight. Data are mean ± SEM from *n* = 5 rats/group.

1 Significantly different from saline (*P* < 0.05).

2 Significantly different from AngII (*P* < 0.05).

### The effect of propranolol on AngII regulation of body weight

**Figure 7:**
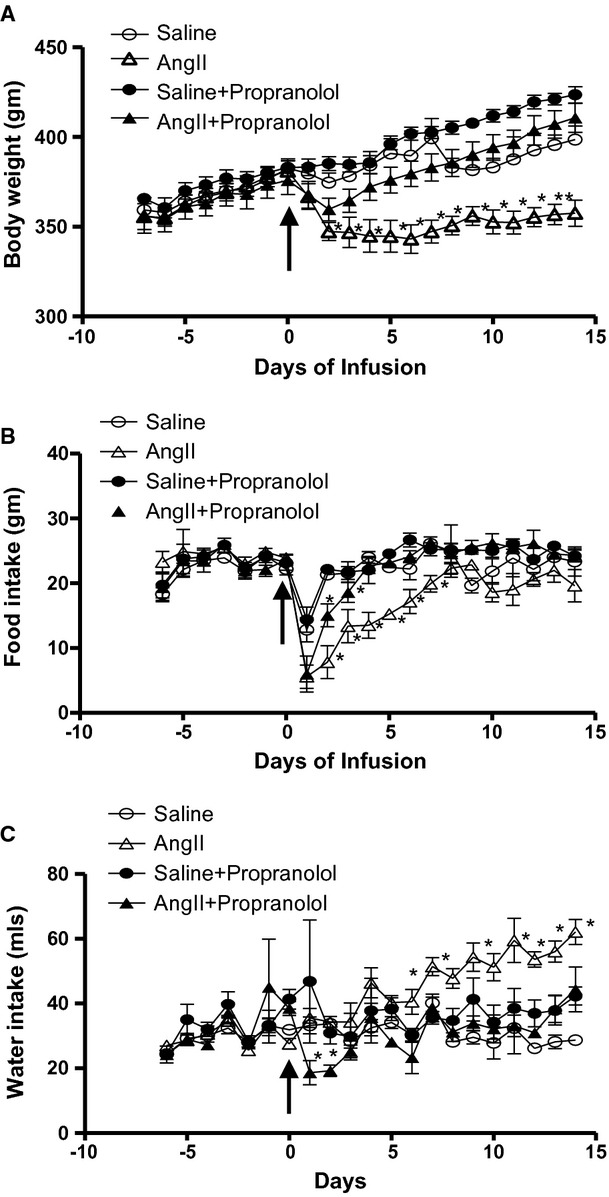
Propranolol administration prevents angiotensin II (AngII) regulation of body weight (A), food intake (B), and water intake (C). Rats were administered saline, saline + propranolol, AngII or AngII + propranolol. Arrow denotes the start of drug treatment by osmotic minipump. Data are mean ± SEM from *n* = 6 rats/group; “*” denotes significantly different from saline (*P* < 0.05).

To determine if AngII-induced increases in sympathetic neurotransmission contribute to AngII-mediated reductions in body weight, the nonselective β-receptor antagonist, propranolol, was administered to rats receiving saline- and/or AngII- (400 ng/kg per minute) for 14 days. Propranolol had no effect on blood pressure in saline-infused rats (data not shown); however, propranolol administration significantly decreased the hypertensive response to AngII (AngII: 125 ± 10; AngII + prop: 102 ± 5 mmHg, *P* < 0.05). AngII infusion decreased body weight beginning on day 3 of infusion (Fig. 7A). Administration of propranolol to saline-infused rats resulted in a modest increase in body weight compared to saline. In rats coadministered AngII + propranolol, AngII-mediated reductions in body weight were eliminated. Moreover, propranolol administration eliminated AngII-induced reductions in food (Fig. 7B) and water intake (Fig. 7C). Propranolol administration abolished AngII-induced increases in plasma concentrations of free fatty acids (saline: 0.13 ± 0.02; AngII: 0.21 ± 0.03; saline/propranolol: 0.11 ± 0.02; AngII/propranolol: 0.08 ± 0.01 mEq/L).

## Discussion

Results from this study support the hypothesis that AngII increases sympathetic neurotransmission to adipose tissue. Elevations in catecholamine turnover in white and brown adipose tissue were demonstrated at doses of AngII that stimulated the turnover of NE in cardiovascular tissues (left ventricle, kidney) and elevated plasma concentrations of free fatty acids. In white adipose tissue, a similar increase in NE turnover was demonstrated in AngII-infused and pair-fed rats, suggesting that reductions in food intake contribute to stimulated NE turnover. In contrast, elevations in NE turnover in the other tissues examined occurred in response to AngII, but not pair feeding, suggesting that AngII elevated catecholamine turnover independent of reductions in food intake. Increases in sympathetic neurotransmission to adipose tissue following chronic AngII infusion occurred at doses of AngII that elevated plasma free fatty acid concentrations and reduced body weight. Moreover, the administration of a β-adrenergic receptor antagonist prevented AngII-mediated reductions in body weight, and AngII-induced elevations in plasma free fatty acid concentrations. Collectively, these results demonstrate that AngII increases sympathetic neurotransmission to adipose tissue, which contributes to AngII-mediated reductions in body weight.

On the basis of previous evidence demonstrating that AngII stimulates sympathetic neurotransmission (Meldrum et al. 1984; Asbert et al. 1992; Kurz et al. 1997; Storgaard and Nedergaard 1997; English and Cassis 1999), we hypothesized that AngII increases sympathetic neurotransmission to adipose tissue. We examined several indexes of sympathetic function in rats chronically infused with AngII, including plasma and tissue NE concentration, NE uptake transporter density, and the tissue-specific turnover of NE. Results from this study demonstrate that chronic infusion of AngII elevated plasma NE concentrations. However, the magnitude of elevation in plasma NE concentrations was more pronounced at lower AngII infusion doses. Our results are in agreement with previous findings demonstrating that chronic infusion of AngII increased plasma NE concentration in a time-dependent manner (Henegar et al. 1995). In addition, our results extend previous findings by demonstrating more pronounced increases in plasma NE concentration at lower (and presumably less pressor) doses of infused AngII. We previously demonstrated that AngII enhanced electrically evoked release of NE from sympathetic terminals innervating adipose tissue (English and Cassis 1999). It is possible that infusion of AngII increased sympathetic activity by facilitating NE release from presynaptic terminals innervating adipose tissue. Alternatively, infusion of AngII may have enhanced central nervous system sympathetic efferent outflow to adipose tissue (Meldrum et al. 1984).

The measurement of plasma NE concentration represents the balance between spillover and clearance of NE in plasma, and thus does not provide tissue-specific information. Therefore, we determined the effect of chronic AngII infusion on tissue-specific indexes of sympathetic activity, including NE turnover and NE uptake transporter binding density. Previous studies demonstrated that acute and chronic in vitro exposure of brain neuronal cell cultures to AngII stimulated [^3^H]-NE uptake (Raizada et al. 1996). To examine the in vivo effect of AngII infusion on the NE uptake transporter, we performed radioligand binding using [^3^H]nisoxetine as a highly selective ligand for the NE uptake transporter (Tejani-Butt 1992). Both the affinity and the density for [^3^H]NIS binding in ISBAT were similar to previously reported values (King et al. 1999). Moreover, in tissues from saline-infused rats, the density of NE uptake transporter sites in ISBAT was approximately 10-fold greater than in LV, demonstrating the dense sympathetic innervation of ISBAT. Our findings of a dose-dependent increase in the density of NE uptake sites in ISBAT are in agreement with previous results from in vitro studies demonstrating that AngII increased the gene transcription and translation of the brain NE transporter (Raizada et al. 1996).

In addition to examining the NE transporter, we measured tissue-specific NE turnover following synthesis inhibition (Brodie et al. 1966; Cassis et al. 1988c). Surprisingly, relatively few studies have assessed NE turnover in chronic AngII-hypertension (Kline et al. 1990; Henegar et al. 1995). We examined the effect of chronic AngII infusion on NE turnover in tissues of cardiovascular and metabolic relevance to the effects of AngII. In the EF, a white adipose tissue primarily involved in the storage of lipids, the effect of AngII was influenced by the time of exposure and by metabolic status. Initially following AngII exposure (i.e., 7 days), the rate of NE decline in white adipose tissue was reduced. We suggest that the initial reduction in the rate of NE decline in EF may have resulted from baroreceptor-mediated reductions in sympathetic outflow to nonessential tissues (i.e., like white adipose tissue) or vasoconstriction and reductions in adipose blood flow in response to a pressor dose of AngII. However, more prolonged exposure to AngII (14 days) increased the rate of NE decline in white adipose tissue, demonstrating an increase in sympathetic drive. Elevations in the NE turnover rate in white adipose tissue following fasting have been previously reported (Migliorini et al. 1997). However, as only one source of white adipose tissue was examined in this study, it is unclear if effects of AngII to increase NE turnover in adipose tissue are uniform. In pair-fed rats, the rate of NE decline was increased to a similar extent as in AngII-infused rats, demonstrating that reductions in food intake contribute to elevations in catecholamine turnover. In this study we provided pair-fed rats with food intake levels of AngII-infused rats in one allotment. It is possible that the timing of food intake may have influenced efficiency of food use as a fuel as well as catecholamine turnover. Notably, infusion doses of AngII that decreased body weight and elevated NE turnover rate were associated with elevations in plasma concentrations of free fatty acids. These results are in agreement with previous findings from AngII-infused rats demonstrating an increase in plasma free fatty acids (Ran et al. 2005) and suggest that AngII-induced sympathetic activation to adipose tissue resulted in systemic mobilization of lipids. Increased sympathetic activity to white adipose tissue with resultant lipid metabolism would be anticipated to contribute to AngII-mediated reductions in body weight.

In ISBAT, chronic AngII infusion progressively increased the rate of NE decline (from day 7 to day 14 of infusions). Results from pair-feeding studies suggest that AngII-mediated increases in the rate of NE decline in brown adipose tissue do not result from reductions in food intake. Surprisingly, AngII infusion resulted in an increase in ISBAT tissue NE concentration and the NE turnover rate. Increased sympathetic activity to brown adipose tissue would be anticipated to increase peripheral energy expenditure (Cassis et al. 1998a, 2002) and contribute to AngII-mediated reductions in body weight.

In the kidney and left ventricle, organs which contribute to AngII regulation of the cardiovascular system, results demonstrate that AngII-mediated increases in sympathetic neurotransmission were independent of food intake. The observed decrease in NE concentration in LV and kidney in response to AngII is in agreement with previous findings following 10 days of i.v. AngII infusion (Kline et al. 1990). However, in previous studies the rate of NE decline in LV and kidney were not significantly altered by 10 days of AngII infusion, leading these authors to conclude that unknown mechanisms contributed to reductions in NE concentration from chronic AngII exposure (Kline et al. 1990). Our results extend previous findings by demonstrating a marked increase in the rate of NE decline in kidney and LV following more prolonged (i.e., 14 day) AngII exposure, which is suggested to serve as the primary mechanism for reductions in tissue NE concentration. The ability of AngII to increase sympathetic nervous system activity to the heart and kidney may contribute to the pathophysiological role of this peptide hormone in hypertension and heart failure.

The ability of propranolol to eliminate AngII-mediated reductions in body weight and food intake mechanistically ties AngII to β-adrenergic receptor stimulation. Rat adipose tissue possesses predominately β1 and β3-adrenergic receptors involved in catecholamine-stimulated lipolysis (Wernze et al. 1978; Nahmias et al. 1991; Granneman 1995). Propranolol has a higher affinity for the β1/β2 adrenergic receptor (pA2 of 8-9; Wilson et al. 1984) than the β3-adrenergic receptor (pA2 of 6.3; Nahmias et al. 1991). We suggest that propranolol inhibited AngII-mediated reductions in body weight and plasma free fatty acid concentrations due to adipocyte β1-adrenergic receptor blockade. However, as a lipophilic β-receptor antagonist, propranolol would gain access to CNS sites important in the control of sympathetic outflow and food intake, potentially contributing to its ability to block the effect of AngII. Alternatively, the ability of propranolol to eliminate AngII-mediated reductions in body weight may relate to its antihypertensive properties; however, previous studies in our laboratory demonstrated that reductions in body weight in response to AngII were independent of blood pressure (Cassis et al. 1998b).

In conclusion, chronic AngII infusion increased the rate of NE decline in adipose tissue, indicative of heightened sympathetic drive. In white adipose tissue, increases in sympathetic neurotransmission by AngII were mediated partially by reductions in food intake. These results support the hypothesis that AngII stimulates sympathetic outflow to adipose tissue for the control of lipid metabolism. The significance of these results relates to disease states associated with increases in systemic or locally formed AngII, including obesity-related hypertension (Yiannikouris et al. 2012), heart failure (Staroukine et al. 1984; Pedersen et al. 1986), and cirrhosis (Wernze et al. 1978; Asbert et al. 1992). Alterations in AngII in these disease states with associated changes in sympathetic neurotransmission may impact not only cardiovascular control but may also modulate lipid metabolism and body weight.
